# Walking a tightrope: Social support in early adulthood in resource-constrained South Africa

**DOI:** 10.1177/02654075251337551

**Published:** 2025-05-22

**Authors:** Dorottya Hoór, Vuyiswa Nxumalo, Nuala McGrath, Janet Seeley, Maryam Shahmanesh, Guy Harling

**Affiliations:** 1 4919 University College London, UK; 2 560159 Africa Health Research Institute, South Africa; 3 University of Southampton, UK; 4 University of KwaZulu-Natal, South Africa; 5 London School of Hygiene of Tropical Medicine, UK; 6 University of the Witwatersrand, South Africa

**Keywords:** social support, personal networks, South Africa, waithood, gender, family, friends

## Abstract

Supportive social relations are crucial to wellbeing as young adults transition to independence, especially when these transitions are impeded by limited employment and educational opportunities, leading to lengthy ‘waithoods’. Yet, there is limited empirical evidence on what young adults’ support networks look like in highly resource-constrained settings. We therefore analysed the core support networks of 929 16-29 year-old rural South Africans to explore their social support landscape, using descriptive statistics and multilevel regression models. We found that these youth have small but intense support networks, with contacts often providing multiple types of support on a daily basis. While kin and parents are important support sources when present, parents are also frequently missing from these networks. Women received more intense support than men with more kin ties; men’s networks contained more friends. While non-kin ties (friends and romantic partners) provide substantial support, they may also be unstable: for young men through dissolution following conflict and for young women due to the transactional nature of romantic relations. These findings imply that where work opportunities are scarce, young people’s support networks are smaller and less parentally focused than elsewhere, potentially increasing their fragility and raising the risk of isolation.

## Introduction and context

Early adulthood, a period of transition from adolescence to adulthood takes place between late teenage years and approximately age 30 (Arnett, 2000). This period is typified globally by opportunity and challenges as young adults complete their education and establish financial and residential independence. These actions represent increasing autonomy and personal responsibility, and are accompanied by experimentation and identity exploration that will define adulthood (Antonucci et al., 2010; Arnett, 2014; Ponti & Smorti, 2019). This period is thus associated with substantial personal strain (Arnett, 2014), which in turn can embed habits that harm health and wellbeing in both the short- and long-run. Throughout the life course, stresses and strains can be mitigated by support from close social relations often with bonds of reciprocity within personal social networks, as highlighted in the Convoy model (Antonucci et al., 2010; Kahn & Antonucci, 1980). However, the role of support networks during this period is less well-explored than other life stages (Antonucci et al., 2010).

Early adulthood in high-income countries is characterised by both continuity and change in support networks (Parra et al., 2015). In terms of supportive relations, parental relationships remain crucial to young adults. Positive parental relations buffer against stress and contribute to young adults’ resilience and self-esteem (Howard Sharp et al., 2017; Lee & Goldstein, 2016; Roberts & Bengtson, 1993). Strong social ties to family and friends can also reduce the negative impact of risk factors, including family and community violence and limited access to resources, by providing emotional and informational support (Pereira et al., 2023). As young adults move out of parents’ homes, relationships with friends gain increased saliency (Collins & van Dulmen, 2006; Tanner, 2006). At these ages, supportive and warm friendships predict positive mental health and buffer against stress (Bagwell et al., 2005; Lee & Goldstein, 2016; Tanner, 2006), while antagonistic friendships predict the reverse (Bagwell et al., 2005). Lastly, young adults typically build longer romantic relationships than adolescents, characterised by mutual emotional support, caregiving, and stronger emotional bonds. As such relations are linked to less loneliness (Lee & Goldstein, 2016), the support role of romantic partners also increases in this period (Furman & Winkles, 2012).

In high-income settings, several gender differences seen in adolescence persist in early adulthood. Young women have more social ties and mobilise more social support in stressful situations than men (Martinez-Hernaez et al., 2016). Young women also feel closer to, and maintain more contact with family members, including parents and siblings, and regard their parents as a more important source of emotional support (Norona et al., 2015; Parra et al., 2015; Sneed et al., 2006). Regarding friendships, young women feel more comfortable discussing loaded topics and disclosing personal information with same-gender friends and interact with them in more affiliative ways, such as talking and sharing stories, while young men tend to engage in more activity-oriented pursuits such as playing sports (Norona et al., 2015). Young women are also more likely to solve conflicts with friends through compromise and considering the needs of both parties, while young men are more likely to opt for solutions that primarily cater to their own needs.

Most enquiry to date into emerging or early adulthood has been in high-income settings, notably the United States. However, as the experience of transition into adulthood is postulated to vary by geography, culture and class, a wider consideration seems crucial (Arnett, 2000). Such enquiry may be particularly important where processes that are normative in high-income settings – higher education, initial employment, relationship stabilization and family building – are less common. Such divergence is the case in many lower- and middle-income countries (LMICs), where historical (including colonial) and ongoing social and economic dislocation means that education is scarce or of poor quality and youth unemployment is very high. Consequently, youth struggle to reach key milestones associated with adulthood elsewhere (Honwana, 2014). This protracted phase of social stagnation between adolescence and adulthood has been observed in many LMICs and is sometimes described as “waithood”, a prolonged and difficult transition from dependent childhood to independent adulthood characterised by economic independence and other social markers of adulthood, e.g., completing education and starting a family (Bernays et al., 2020; Ngwenya & Ross, 2024).

Waithood has substantial implications for LMIC youths’ social support systems. Youth necessarily improvise livelihoods in the limbo of informal or no employment as they invent new forms of interaction with society and new ways of existing in the margins (Levy & Dubinsky, 2023). They are often compelled to ‘hustle’ to create new forms of livelihood, manage risks and survive on the margins; this typically entails informal employment, which may include transactional sex (Ngwenya & Ross, 2024; Thieme, 2017). The multiple and intersecting uncertainties associated with waithood may also shape their aspirations, e.g., to move away from rural homes (Ngwenya et al., 2023). As a result, their personal relations also develop outside of dominant economic and familial frameworks and encompass a wide range of social contacts, including siblings, extended family, mentors, church members and neighbours (Soji, 2018). However, due to economic and demographic homophily, these networks may provide young people with fewer embedded resources (e.g., valuable information, connections, material resources), limiting their ability to achieve their goals (Lin, 2001). Although local economic and political challenges can significantly hamper youths’ efforts to forge alternative paths to adulthood and to obtain adequate support, the support they do get from relatives and friends is believed to reduce the loneliness of this period and protect them against various risk factors (Levy & Dubinsky, 2023; Martinez-Hernaez et al., 2016). Yet evidence of how waithood impacts young people’s social support networks is scant.

We therefore aim to explore the support landscape for young people in early adulthood in the highly resource-constrained setting of rural KwaZulu-Natal, South Africa. The setting of rural KwaZulu-Natal is particularly apposite for examining the impacts of waithood on young people’s support networks, as it has few formal jobs and very limited opportunities for self-employment or informal wage work and high rates of out-migration (Statistics South Africa, 2016). It is also characterised by complex, fluid household arrangements involving mobility between households and multiple parallel household memberships, multiple and skipped generations, child-headed households, and the dispersal of children and migrant parents across several locations (Bennett et al., 2015a, 2015b; Hosegood & Timaeus, 2006; Reynolds, 2015). Such social arrangements, compounded by late and decreasingly common marriage, high HIV prevalence and incidence, and orphanhood increase young people’s vulnerability to psychosocial problems and emphasises the need for supportive social networks (Chimbindi et al., 2018; Hill et al., 2008; Hosegood et al., 2009; Mejia-Pailles et al., 2020).

## Literature review

The limited existing evidence suggests that the transition to early adulthood in LMICs is likely to greatly impact support networks, potentially even more than elsewhere. First, LMIC youth might have limited social support in general, with small networks providing limited support. While social support is essential for households experiencing financial hardship, evidence shows that these networks might not be able to provide adequate support (Lubbers et al., 2020). While reciprocity is traditionally seen as a force of integration, in resource-constrained settings, it can also be a burden and source of relational stress, as individuals find it difficult to fulfil obligations of reciprocity when they have limited resources with which to do so (Offer, 2012). As a result, prolonged network mobilisation might lead to relational strain and long-term conflict (Lubbers et al., 2020). Existing evidence in KwaZulu-Natal does suggest that support networks for youth indeed tend to be small and provide limited support (Durevall et al., 2023; Harling et al., 2018).

Second, the specific context of rural South Africa is likely to lead to compositional differences compared to other contexts. On the one hand, young adults in KwaZulu-Natal might struggle to obtain crucial parental support. The high prevalence of single-mother households (Jacobs & Andrews, 2021), absent or unacknowledged fathers (Denis & Ntsimane, 2006; Malherbe & Kaminer, 2022), and the loss of parents to HIV (Hill et al., 2008; Mejia-Pailles et al., 2020; Ngwenya et al., 2023) mean that many young people only know one living biological parent. High rates of labour migration within South Africa further fragment families, with many parents not sharing a physical household with their children (Bennett et al., 2015a; Bennett et al., 2015b). This has important implications since greater geographical distance typically predicts less support exchange (Mulder & van der Meer, 2009). Regarding parental support, most previous studies show that fathers in KwaZulu-Natal provide very limited support to their children (Denis & Ntsimane, 2006), although there is some evidence that this reflects a failure to recognize emotional and financial inputs (Montgomery et al., 2006). Many young people do not receive maternal help either (Harling et al., 2018).

On the other hand, waithood might also lead to a more kinship-based support network. This kinship support extends well beyond the nuclear family, a term that has historically limited valence in the regional context of highly flexible kinship networks (Barbarin & Richter, 2001; Hosegood & Timaeus, 2006; Reynolds, 2015). For instance, ethnographic work on traditional Zulu kinship highlights the importance of wider kin relations, where young people call all their maternal aunts “mother” and paternal uncles “father” (Gluckman, 1987; Krige, 1950). Contemporary Southern African households are also highly fluid and adaptable domestic arrangements, with frequent mobility of children between households and membership in multiple households, suggesting that children with multiple household membership might be able to access more resources through their kin (Hosegood et al., 2005; Parikh et al., 2007; Reynolds, 2015).

Additionally, norms of reciprocity vary by tie type, creating a set of tie-specific expectations, obligations and types of transactions that are considered appropriate (Lubbers et al., 2020; Portugal, 2009). Kinship ties are generally characterised by a strong obligation of bi-directional assistance and deferred reciprocity, transforming help into a “long-term credit”, where recipients are not immediately expected to reciprocate support (Curry et al., 2019; Portugal, 2009). In contrast, friendship ties are based on a narrower norm of short-term reciprocity with less obligation to help, making them a less reliable source of support and more prone to exhaustion and conflict (Nelson, 2000). Amongst 9–16 years old Zulu children, grandparents were primary caregivers for 56% of orphans and 43% of non-orphans (Parikh et al., 2007). Similarly, preliminary evidence from our setting suggests that relatives were the core of young adults’ support networks – evenly split between same-generation and older generations – while friends provide mostly social companionship (Harling et al., 2018).

Finally, waithood may increase gender differences in support networks both in terms of configuration and support flows. While all young people in waithood might delay marriage (Honwana, 2014; Hosegood et al., 2009), young women may remain in the parental household longer – as seen worldwide due to greater family obligation (Fuligni & Pedersen, 2002), and locally due to the combination of normative early motherhood and very limited paternal involvement requiring reliance on family support (Maharaj, 2022). These factors may increase the ratio of kin to friendship ties above that seen elsewhere. Furthermore, young women commonly obtain financial, practical, emotional and informational support from older romantic partners and in part from older relatives (Bernays et al., 2020; Gillespie et al., 2022; Harling et al., 2018; Hunter, 2010). Young men receive emotional support from a wider range of sources, while older or same-generation relatives are the main providers of informational, financial and physical support to them (Harling et al., 2018).

In sum, even though the experience of waithood is likely to impact the support networks of emerging adults, there is limited empirical evidence on what young adults’ support networks look like in highly resource-constrained settings, including those where waithood is normative. We therefore aimed to explore the support landscape for young people in early adulthood in rural KwaZulu-Natal. Based on previous work, we formulate three hypotheses regarding these networks. First, we expect young people in rural KwaZulu-Natal to have small networks and limited access to social support in general. Second, we also expect these networks to have a high prevalence of kin ties, but with limited parental support. Lastly, we also expect to see gender differences, with young women having more kin and romantic partner support than young men. To assess these hypotheses, we analysed data from the baseline round of Sixhumene (“we are connected” in isiZulu), a longitudinal study examining social support networks and sexual health of young people in rural KwaZulu-Natal, South Africa (Nxumalo et al., 2022). By doing so, we provide novel empirical evidence not only on how waithood might impact emerging adults’ support networks but also more broadly on how the experience of transition into adulthood may vary by geography, culture, and class in resource-constrained settings.

## Methodology

### Research design and data collection

This research was conducted within the Health and Demographic Surveillance Site (HDSS) managed by the Africa Health Research Institute (AHRI) in Umkhanyakude District Municipality, in northeastern KwaZulu-Natal. Since 2000, AHRI has conducted semi-annual rounds of demographic and health surveillance on a population of approximately 11,000 households composed of 90,000 resident and non-resident individuals in a bounded area of 438 km2 with the primary mission of measuring the long-term impacts of HIV/AIDS and conducting research towards HIV prevention, treatment, and care. According to latest figures, Mtubatuba Municipality had 42.8% unemployment rate and high dependency ratio, with 47% of households headed by females and the majority of the population being young and dependent (Statistics South Africa, 2011).

Data collection took place in three geographically defined communities as part of Sixhumene, an open-cohort longitudinal study of social networks and sexual health (Nxumalo et al., 2022). We used baseline data from Sixhumene, collected in 2022-23. Participants were recruited in two stages, using a one-step snowball approach. First, the 250 individuals aged 16-24 living nearest to a central point in each community were identified from the most recent AHRI HDSS surveillance round. All eligible individuals were approached either in person at their homes or by telephone on numbers previously provided to AHRI. They were invited to interview, with the expectation of enrolling 200 into the study. Where fewer than 200 participants were enrolled per community, the circle was widened to invite additional 16–24 year olds. Anyone aged 16 or older whom these participants named as a contact and who resided in the HDSS area was invited to enrol in stage two.

Participants’ support networks were measured using an exchange-based personal network approach. Network members were elicited in name generator questions covering emotional, practical, informational, financial and socialisation support, as well as conflict and recent sexual contact (Supplemental Material 1). Name interpreter questions were then asked for each named contact (“alter”) to elicit alter characteristics, including demographics, type and length of relationship to ego, residency and length of relationship to the respondent (“ego”). Frequency of support provision (and conflict) from each alter was also recorded on a six-level scale based on interaction over the past six months (1-less than once a month, 2-once a month, 3-few times a month, 4-once a week, 5-few times a week, 6-everyday/almost every day). The Sixhumene interview also collected information about ego’s perceived level of social support, as well as socio-demographics, sexual behaviour and risk factors for poor sexual health outcomes. Interviews were conducted in isiZulu by a team of experienced, locally resident interviewers. Sixhumene was discussed with AHRI’s Community Advisory Board prior to finalization, and they provided formal approval during the ethical review process. The study was approved by the University of KwaZulu-Natal’s Biomedical Research Ethics Committee (0359/2019), University College London Research Ethics Committee (15231/009), and by the KwaZulu-Natal Department of Health (KZ_201911_012).

### Measures

We computed several personal network characteristics. Overall network size was calculated as the number of alters providing at least one kind of support monthly; network size for each support type was similarly calculated as the number of alters providing a given type of support at least monthly. We used the five binary measures of positive support provision (excluding conflict) to compute tie multiplexity, i.e., the average number of different support types received from the same alter (McCarthy et al., 2019), and calculated gender homophily as the proportion of same-gender alters among those providing at least monthly support. To account for complex, fluid, geographically-dispersed and extended family and household structures, we categorised alters according to their kinship, residency and relationship to egos: (1) co-resident mother; (2) co-resident father; (3) non-co-resident mother; (4) non-co-resident father; (5) co-resident other kin; (6) non-co-resident other kin; (7) non-sexual non-kin (hereafter, friends); and (8) romantic and sexual partners.

Frequency of support was measured both at the ego and at the tie level. We re-coded the categorical variables of support frequencies as the approximate number of days per month on which an alter gave support to an ego. Accordingly, we valued support received “less than once a month” as 0, “monthly” support as 1, “few times a month” as 2, “weekly” as 4, “few times a week” as 10, and “daily/almost daily” as 30. These frequencies were calculated at the tie level for each support type (including conflict), and then summed within the five positive support types to calculate a total ego-level quantity of support received (i.e., values exceeding 30 imply that there were days when they received support from more than one person). To assess whether the level of received support matched participant needs, we used the eight-item Duke-UNC Functional Social Support questionnaire capturing sufficiency of support received on a five-point scale (from ‘As much as I like’ to ‘Much less than I would like’) (Broadhead et al., 1988). We summed responses into an index ranging from 8 to 40, with higher values indicating more perceived support.

### Data analysis

First, we described respondent demographics and checked for differences by ego gender using *t* tests for continuous variables and chi-squared tests for categorical and count variables. We also stratified by age (16–20, 21–29) to see how late teenagers and young adults differed. We described common combinations of support types provided by alters visually using an UpSet plot (Conway et al., 2017). We then described ego’s networks in terms of alters’ relationship categories, expressed as absolute size and proportions. We also computed the proportion of participants who received any kind of monthly support from parents by residency status, and overall monthly support frequency by type of support.

To understand tie-level support provision patterns, we conducted multilevel ordinal logistic regression (alters nested within egos) for monthly support of each type (including conflict), treating ego-alter relationship type as our explanatory variable. We also included network size, ego gender, ego-alter age difference and ego age as potential confounders. Finally, to evaluate whether our primary findings differed by ego’s gender, we added interaction terms between ego gender and ego-alter relationships and tested for effect-modification using Wald tests. All analysis was conducted in Stata v17 (StataCorp; College Station, TX).

## Results

We sampled 944 individuals aged 16–24, of whom 158 (16.7%) were no longer resident in the study areas by the time the study team sought them, 58 (6.1%) were unreachable after three contact attempts and 64 (6.8%) declined to participate. The 664 interviewed individuals named 2127 alters unique within their interview, of whom 684 (32.2%) were aged 16–29 and resident in the HDSS area. Among these, 164 were already enrolled in the study (i.e., duplicates) and 17 others were unreachable (2 deceased, 11 unknown at stated home, 2 unable to communicate and 2 were not referred by their ego). Of the remaining 503, 100 (19.9%) were unreachable and 117 (23.3%) declined to participate, leaving 286 stage two respondents (i.e., named contacts of the initial sample). Nineteen interviews were lost due to a tablet malfunction, leaving a total sample of 931 respondents aged 16–29. Two individuals were excluded from our analysis – one had missing gender information, the other named no contacts. The 929 remaining respondents named 3023 alters unique within their interview through the seven name generator questions.

**Figure 1.**
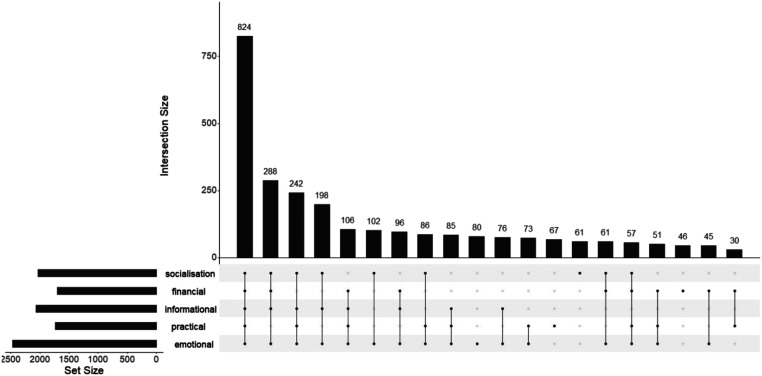
Upset plot of support multiplexity.

Of the 929 respondents, 502 (54.0%) were female and their mean age was 19.8 years. All participants were Black African. Reflecting typical characteristics of waithood, almost all respondents lived with their family, were unmarried and unemployed – employment referring to any kind of formal or informal paid activity – with only about half still in education (Table 1). Among respondents aged 20–29 68.6% were neither in education nor had any kind of employment. Respondents named 3023 alters in total, who were on average 9.5 years older than them, and thus had a mean total network size of 3.1, with specific support types ranging between 1.8 and 2.7 for positive support and 0.6 for conflict (Table 2). Network sizes were similar by gender, except for a slightly larger financial support network for women (1.9 vs. 1.7, *p* = .007). Mean tie multiplexity, the number of different types of support provided by each alter, was 3.3; women had a slight but statistically significant higher level of multiplexity (3.4 vs. 3.2, *p* = .013). The most common combination of support ties was all five types of support provided (27% of all alters). Most other common combinations were built on a base of emotional and information support, with single-support ties being very uncommon (Figure 1). Co-resident mothers had the highest mean tie multiplexity, providing 4.1 types of support, followed by co-resident fathers (3.6), other co-resident family members (3.5), romantic partners (3.2) and friends (3.1). Patterns looked similar across ages when we stratified egos above/below age 20, with support levels dropping even further in the older cohort (Supplemental Material 4).

**Table 1.** Demographic characteristics.

	Total	Male	Female	*p*-value
	*N* = 929	*N* = 427 (46.0%)	*N* = 502 (54.0%)	
Age on interview date	19.8 (3.2)	19.5 (3.0)	20.1 (3.3)	0.008
Lives with family	908 (97.7%)	420 (98.4%)	488 (97.2%)	0.24
Unmarried	926 (99.9%)	427 (100.0%)	499 (99.8%)	0.36
Currently in school	477 (51.4%)	251 (58.9%)	226 (45.1%)	<0.001
Highest completed education				<0.001
Primary school	23 (2.5%)	13 (3.0%)	10 (2.0%)	
Secondary school	571 (61.5%)	287 (67.2%)	284 (56.7%)	
Matriculation	298 (32.1%)	120 (28.1%)	178 (35.5%)	
Certificate	13 (1.4%)	5 (1.2%)	8 (1.6%)	
Diploma	13 (1.4%)	2 (0.5%)	11 (2.2%)	
Bachelors degree	9 (1.0%)	0 (0.0%)	9 (1.8%)	
Honors, masters or higher	1 (0.1%)	0 (0.0%)	1 (0.2%)	
Employment status				<0.001
Full-time	14 (1.5%)	7 (1.6%)	7 (1.4%)	
Part-time	81 (8.7%)	60 (14.1%)	21 (4.2%)	
Not working	833 (89.8%)	360 (84.3%)	473 (94.4%)	

Data are presented as mean (SD).

**Table 2.** Ego-level characteristics.

	Total	Male	Female	*p*-value^a^
	*N* = 929	*N* = 427	*N* = 502	
Network size				
Overall	3.1 (1.36)	3.1 (1.46)	3.1 (1.28)	0.61
Emotional	2.7 (1.56)	2.6 (1.64)	2.7 (1.49)	0.11
Practical	1.9 (1.28)	1.9 (1.30)	1.9 (1.27)	0.78
Informational	2.2 (1.65)	2.1 (1.74)	2.3 (1.57)	0.15
Financial	1.8 (1.37)	1.7 (1.37)	1.9 (1.35)	0.007
Social companionship	2.2 (1.58)	2.1 (1.65)	2.2 (1.53)	0.39
Conflict	0.6 (1.03)	0.6 (1.05)	0.5 (1.01)	0.21
Multiplexity	3.3 (1.16)	3.2 (1.19)	3.4 (1.14)	0.013
Gender homophily (% of same-gender alters)	68.4%	57.6%	77.7%	<0.001
Network composition (mean proportion of networks)				
Friend	30.9%	35.0%	27.4%	<0.001
Co-resident mother	17.8%	16.0%	19.3%	0.025
Mother outside household	3.8%	4.0%	3.7%	0.65
Co-resident father	2.1%	2.8%	1.5%	0.022
Father outside household	2.0%	2.6%	1.4%	0.027
Co-resident other family	21.0%	19.3%	22.4%	0.078
Other family outside household	11.8%	12.3%	11.4%	0.55
Romantic partner	10.7%	8.1%	12.9%	<0.001
Parental support				
Any parent (resident or not)				0.003
none	367 (39.5%)	178 (41.7%)	189 (37.6%)	
one parent	465 (50.1%)	192 (45.1%)	273 (54.4%)	
both parents	97 (10.4%)	57 (13.3%)	40 (8.0%)	
Co-resident parents				0.004
none	448 (48.2%)	221 (51.8%)	227 (45.2%)	
one parent	447 (48.1%)	184 (43.1%)	263 (52.4%)	
both parents	34 (3.7%)	22 (5.2%)	12 (2.4%)	
Co-resident mothers	451 (48.5%)	188 (44.0%)	263 (52.4%)	0.011
Co-resident fathers	64 (6.9%)	40 (9.4%)	24 (4.8%)	0.006
Mother outside household	115 (12.4%)	58 (13.6%)	57 (11.4%)	0.30
Father outside household	63 (6.8%)	36 (8.4%)	27 (5.4%)	0.065
Mother (co-resident and not)	532 (57.3%)	230 (53.9%)	302 (60.2%)	0.053
Father (co-resident and not)	127 (13.7%)	76 (17.8%)	51 (10.2%)	<0.001
Monthly support frequency				
Emotional	54.5 (49.4)	50.3 (50.7)	58.1 (48.1)	0.016
Practical	33.3 (30.7)	30.1 (30.0)	36.1 (31.1)	0.003
Informational	33.5 (38.4)	29.4 (37.0)	37.1 (39.3)	0.002
Financial	20.5 (25.7)	19.1 (24.0)	21.7 (27.1)	0.12
Social companionship	35.1 (38.3)	37.8 (41.0)	32.8 (35.6)	0.049
Conflict	6.1 (16.2)	6.5 (17.3)	5.8 (15.2)	0.56

Two individuals are dropped in this table – one had no gender specified, the other named no alters.

Data are presented as mean (SD) for continuous measures, and *n* (%) for categorical measures.

^a^Tests of difference are *t* tests for continuous variables and chi-squared tests for categorical and count variables.

Perceived adequacy of support was high: almost one-third (274, 30%) perceived receiving all forms of support as much as they would have liked, while 734 (79%) reported a score higher than 32, i.e., above all support types being “nearly as much as wanted” (Supplemental Material 2).

While all respondents named more same-gender alters (mean 68%), this tendency was stronger among women (78% vs. 58% for men; Table 2). Friends comprised the largest proportion of support networks (31%), followed by other co-resident family members (21%), co-resident mothers (18%), other non-co-resident family members (12%) and romantic partners (11%). Fathers, whether co-resident or not, and non-co-resident mothers were rarely named. While most of these patterns were similar for men and women, men’s networks had higher proportions of friends (35% vs. 27%) and fathers (5.4% vs. 2.9%), while women had a higher proportion of romantic partners (13% vs. 8%) and co-resident mothers (19% vs. 16%).

Two-fifths of participants (39.5%) received no monthly support from either parent and only 10.4% regularly received support from both; these values are even lower when restricted to the same household (Table 2). Mothers provided support to many more participants (57.3% vs. 13.7% for fathers), a gap that was even wider for co-resident mothers and fathers (48.5% vs. 6.9%). While maternal support was far more common for both males and females, fathers more commonly supported young men (17.8% vs. 10.2% for women), who consequently more often received support from both parents (13.3% vs. 8.0%). Amongst kinship-based support ties, support from same generational kin (siblings and cousins) was the most common type (49.2%), followed by parental support (44.0%). Support from other sources (parental generation non-parental kin and generation of grandparents) were negligible (3.1% and 3.7%).

Respondents reported receiving emotional support most often, from approximately two people per day on average, followed by social companionship, practical and informational support roughly once per day. Financial support was less common and conflict arose roughly six times per month (Table 2). Women received emotional, practical and informational help significantly more often than men, with equal levels of other support and conflict. Patterns of support at the alter level were similar to those at the ego level, reflecting similar network sizes for men and women for each support type with women receiving most support types slightly more frequently with exception of social companionship on a per tie basis (Supplemental Material 4).

Among all alters named, co-resident mothers provided the most frequent support of all kinds, but were also the ties with the most conflict (Table 3). Other important sources of emotional support were romantic partners, fathers (when present) and other co-resident family, the latter also providing substantial informational support. Practical support receipt was very strongly associated with co-residence. Financial support appears to come from a wide range of social contacts, with friends being the least frequent providers. Friends and co-resident family (including parents) were the most common providers of social companionship. Finally, women received significantly more emotional and informational support per alter, and also engaged in less conflict.

**Table 3.** Multilevel ordinal logistic models of support frequencies.

	Emotional	Practical	Informational	Financial	Social companionship	Conflict
Relationships (vs friend)						
Co-resident mothers	5.09 [3.37, 7.69]	39.9 [27.3, 58.2]	5.44 [3.81, 7.75]	5.66 [4.05, 7.93]	1.84 [1.29, 2.63]	8.59 [4.62, 16.0]
Co-resident fathers	2.40 [1.14, 5.10]	17.0 [9.18, 31.5]	1.57 [0.81, 3.05]	2.34 [1.28, 4.29]	0.99 [0.50, 1.94]	2.79 [0.94, 8.26]
Mothers outside household	1.06 [0.62, 1.85]	0.70 [0.42, 1.15]	1.13 [0.69, 1.85]	2.33 [1.48, 3.68]	0.10 [0.06, 0.17]	0.35 [0.14, 0.86]
Fathers outside household	0.55 [0.28, 1.11]	0.47 [0.23, 0.94]	0.77 [0.40, 1.46]	2.20 [1.23, 3.93]	0.06 [0.03, 0.11]	0.17 [0.05, 0.65]
Other co-resident family	1.91 [1.40, 2.61]	25.0 [18.6, 33.5]	2.51 [1.92, 3.28]	1.68 [1.30, 2.17]	1.42 [1.08, 1.85]	1.46 [0.90, 2.37]
Other family outside household	0.74 [0.53, 1.03]	1.13 [0.85, 1.50]	1.02 [0.76, 1.37]	1.23 [0.92, 1.64]	0.23 [0.17, 0.32]	0.41 [0.23, 0.73]
Romantic partners	1.52 [1.08, 2.15]	0.37 [0.26, 0.52]	1.16 [0.86, 1.55]	2.13 [1.60, 2.84]	0.34 [0.26, 0.45]	1.42 [0.82, 2.46]
Ego network size	2.27 [1.95, 2.65]	1.09 [1.01, 1.18]	1.77 [1.57, 2.00]	1.21 [1.11, 1.32]	1.74 [1.56, 1.95]	0.70 [0.57, 0.86]
Ego-alter age difference (per year)	1.00 [0.99, 1.01]	0.99 [0.98, 1.00]	1.01 [1.00, 1.02]	1.05 [1.04, 1.06]	0.99 [0.98, 1.00]	1.02 [1.00, 1.03]
Ego age (per year)	0.97 [0.90, 1.03]	1.03 [0.99, 1.07]	1.05 [1.00, 1.12]	1.01 [0.97, 1.05]	0.99 [0.95, 1.04]	0.93 [0.85, 1.01]
Ego female (vs male)	1.80 [1.20, 2.70]	1.04 [0.83, 1.30]	1.79 [1.29, 2.49]	1.22 [0.96, 1.56]	0.83 [0.61, 1.12]	0.53 [0.31, 0.92]
Ego N	927	924	927	923	926	927
Alter N	2986	2974	2984	2974	2985	2987

All models are multilevel models with ego-level random intercepts. Values are Odds Ratios and [95% Confidence Intervals].

When we considered how our findings differed by ego’s gender, we found that women received emotional support more frequently from friends and other non-co-resident family (often siblings, aunts and cousins) (Supplemental Material 5). They also received practical support more frequently from other co-resident family, but less frequently from romantic partners. Women received informational support more frequently from several sources, including co-resident mothers, other co-resident family members and friends, as well as financial support from romantic partners. Women received less companionship from friends than men but also had less frequent conflict with friends and romantic partners.

## Discussion

In this analysis of the social support landscape of 929 young adults in resource-constrained rural KwaZulu-Natal, while some of our prior expectations were confirmed, others were contradicted. On the one hand, in line with our prior expectations we found that young adults had small networks, characterised by the high prevalence of kin and friends, and often lacked support from parents. On the other hand, these networks were also characterised by highly multiplex support ties and frequent support provision, frequently providing an adequate level of support to young people.

First, we expected local youth to have limited support from a few network members. We did find that they have smaller support networks than reported in other settings (Neal, 2025), with an average of only 3.1 network members. However, in contrast to existing research (Durevall et al., 2023; Harling et al., 2018; Lubbers et al., 2020), these smaller networks appeared to provide most young people with broadly adequate support through intense support relations, characterised by highly multiplex ties and frequent support provision at both the ego and tie-level. Ties on average provided 3.3 different kinds of support and respondents received on average at least daily emotional, informational, practical and social companionship support.

Such intense support provision by a handful of key social contacts might be crucial to ensure that young people meet their support needs, but may also endanger the stability of support over time. In a setting where resources are scarce, individuals may be unable to fulfil obligations of reciprocity, resulting in relational strain or loss (Lubbers et al., 2020; Offer, 2012; Portugal, 2009). Increased interpersonal conflict risks negatively impacting wellbeing of youth and increasing tie dissolution – endangering the long-term stability of these small but intense support networks and contributing to youths’ sense of uncertainty (Asselberg, 2016). Furthermore, as Offer (2012) argues, intense support provision in resource-constrained settings may lead to social fragmentation more generally, as the inability to reciprocate support leads to individuals being excluded or withdrawing from supportive social networks. Ethnographic work elsewhere in South Africa highlights a lack of support for and community engagement with youth due to community values expecting them to reciprocate support provision, which they cannot fulfil (Soji, 2018). Lastly, these small networks may also be prone to instability due to geographic mobility, as many young people facing uncertainty in these rural communities chose migration to access better opportunities and fulfil their aspirations (Ngwenya et al., 2023).

Second, we expected young people to have limited access to parental support and other kin to be important support providers. Our results reinforce key findings of earlier research on the fragmentated nature of nuclear families alongside an increased involvement of other kin. Parents in general, and fathers in particular, are often missing from youths’ support networks. Strikingly, almost 40% of respondents did not receive any kind of support from either parent, and only 10% received support from both, with only 14% of respondents reporting any kind of paternal support, and only 7% reporting a co-resident father. This paints an even more dire picture of paternal support than those reported by Denis and Ntsimane (2006), where 27% of fathers resided with their children and 34% provided regular support.

Such limited parental involvement may be explained by a combination of factors, including deceased and unacknowledged parents, as well as known living parents failing to provide support. The latter is both explained and exacerbated by the geographical dispersion of families in KwaZulu-Natal, which prohibits active day-to-day support provision (Mulder & van der Meer, 2009). As a result, non-coresident parents were substantial providers only of financial support to young adults. While previous research suggests that grandparents or other parental generation kin (e.g., aunts and uncles) might take on a primary caregiver role to children (Hosegood & Timaeus, 2006; Parikh et al., 2007), they appear to provide only negligible direct support to young adults in our data. These results are particularly alarming considering the established importance of parental support for the psycho-social well-being of young adults (Howard Sharp et al., 2017; Lee & Goldstein, 2016; Pereira et al., 2023), as well as our own findings that co-resident parents, especially mothers, are major providers of all kinds of support. Lastly, in line with research in other settings, parental ties, particularly to co-resident mothers had the most conflict, potentially resulting in so-called ‘conflicted support’ and relational ambivalence (Fingerman et al., 2013; Hoór & Bellotti, 2025). Conflicted or limited access to parental support might make young adults more vulnerable to the several of risk factors associated with waithood.

Wider kinship-based support ties were extremely important for these young adults. Family members beyond parents – in particular, same-generational kin, such as siblings and cousins – were frequent sources of emotional, practical, informational and financial support and provided a larger portion of support networks than in high-income countries (Amati et al., 2015; Bidart et al., 2020). Such ties may have been retained beyond the ages which they are typically central to youths’ lives in higher-resource settings due both fewer venues through which to make non-kin connections (e.g., tertiary education, workplaces) and to their relative stability underpinned by a strong sense of obligation and deferred reciprocity (Lubbers et al., 2020; Portugal, 2009).

While kin were important, one-third of regular support sources to these youth were non-romantic non-kin, who frequently provided several types of support. This finding aligns with research elsewhere, highlighting the prominence of friendship ties amongst young adults (Collins & van Dulmen, 2006; Tanner, 2006). These friendship ties can be expected to positively contribute to the wellbeing of young adults insofar as supportive friendships are linked to better mental health (Bagwell et al., 2005; Tanner, 2006), in part by buffering against stress (Lee & Goldstein, 2016). Friendship ties, however, are typically more vulnerable to relational strain and tie dissolution, especially if heavily drawn-upon in the resource-scarce environment of waithood, as they carry more limited obligations and norms of more immediate reciprocity than family ties (Nelson, 2000).

Lastly, we found gender differences observed in other settings reproduced in ours. In line with existing evidence, young women in Sixhumene received most kinds of support more often than young men – although not from many more sources – and their support ties were more often gender-homophilous. Women’s networks also contained more kinship ties, and they received more of most support types from non-parental family members, echoing earlier work showing that young women are often closer to their families and regard them as more important support resources (Harling et al., 2018; Norona et al., 2015; Parra et al., 2015; Sneed et al., 2006). In contrast, young men reported significantly more friends in their network. However, these friends were more likely to engage these men in conflict than women’s friends, who provided more emotional support. These patterns align with research elsewhere, suggesting that waithood may not greatly change how youth interact in gendered ways with their friends (Norona et al., 2015).

The observed gender differences in network composition – young women reporting more family members and young men more friends – might also generate differences in network stability over time, if kin ties are more durable than those of friendship ties. If such churn has negative implications for health and wellbeing, additional support for young men may be needed, for example through interventions to build or strengthen their ties to family and friends.

Finally, echoing earlier ethnographic work in KwaZulu-Natal (Hunter, 2009, 2010), we find that young women are more likely to receive financial support from romantic partners, which may pose additional risks to young women. Young women receiving financial support from romantic partners do not only reflect the “mutually constitutive nature of intimacy and exchange” (Hunter, 2009, p. 136) but also highlights how young men and women might employ different coping strategies to overcome financial hardship and labour market inequalities in rural South Africa’s resource-constrained setting. Young women in resource-poor households may create “disposable ties” to obtain support from alternative sources (Desmond, 2012). These relationships are characterised by accelerated development of intimacy and provision of all kinds of support and might be short-lived, breeding instability and misgivings amongst peers (Desmond, 2012; Lubbers et al., 2020). Amongst young Zulu women and their partners, love is often entwined in a set of reciprocal obligations, which may include monetary gifts, affection, emotional support, domestic labour and sex. As these sets of obligations are intertwined in real feelings and evolve over time, violating these obligations may cause stress or the dissolutions of these relationships, negatively impacting women (Hunter, 2009). Finally, such sexual relationships between young women and higher-resource partners also increases women`s vulnerability to acquiring sexually transmitted infections, including HIV (Ranganathan et al., 2017; Sprague et al., 2022).

### Strengths and limitations

This study has the strength of drawing a representative sample of young people from a well-described population, and capturing a detailed description of the social lives of almost 1000 young people. It also, however, has potential limitations. First, this analysis is from a specific rural South African setting with particularly high levels of youth unemployment and people living with HIV. While precise support dynamics will differ by context, waithood is not exclusive to South Africa or even LMICs: social and economic uncertainty leaves young people globally grappling with restricted futures (Honwana, 2014). Replicating this analysis elsewhere will help clarify where specific economic, social and cultural contexts modify young adults’ support networks, and how social networks and precarious transitions to adulthood affect one-another.

Second, our data are self-reported and egocentric. The self-reported nature of the data does not allow validation of egos’ responses against those of their alters, which makes the data vulnerable to social desirability bias among other concerns. This concern would be heightened if this bias varied by ego or alter characteristics. Similarly, even though participants generally report receiving enough support on the Duke-UNC Functional Social Support scale, because the scale measures support relative to desired or expected levels we cannot rule out the possibility that young adults in our study still receive less support in absolute terms than would be considered acceptable – or actually experienced – elsewhere. This would be particularly likely if support sources in this rural South African setting are limited, as appears to be the case. Furthermore, the egocentric data design does not allow evaluation of the effects of broader societal processes, such as social fragmentation. Future work to generate sociocentric networks in Sixhumene should allow cross-validation of information reported by both sides of each relationship and also offer a better understanding of local community structures. 

Third, using cross-sectional data only captures a snapshot of support networks. Social networks are naturally dynamic over time, and the intensive support provision reported here in a resource-constrained setting may lead to important changes in network structure and content. Sixhumene’s longitudinal design addresses this problem and will allow exploration of these changes in the future. Finally, we did not measure respondents’ gender identity, sexual orientation or disability; these aspects of participants lives could be included in future research.

## Conclusion

Supportive social relations are crucial to the wellbeing of young adults. We find that when early adulthood becomes a waiting time, traditional support channels may become disrupted, and young people’s support networks may be compositionally reconfigured. The personal social networks of rural South African youth that we describe are small, highly multiplex, yet most youth perceive them as providing adequate support. Besides a significant proportion of friends, they also retain a large proportion of same generational kin – while many parental ties (especially paternal ones) are absent. These patterns might reflect the effects of waithood both in maintaining kin ties and retarding the development of new ties through new residential and professional opportunities. As obligations of reciprocity in resource-scarce environments might strain and exhaust existing support ties, friendship ties might become overstretched by the multiple support types they are asked to sustain. In contrast, kinship ties may provide temporal stability, as they are often characterised by a more long-term understanding of reciprocity. The higher proportion of young men’s ties to, support from, and conflict with non-kin suggests that they may have particularly fragile support networks in this setting. Interventions to support existing support ties and expand opportunities, especially for young men, may be vital in settings where opportunities for social growth are limited.

## Supplemental Material

Supplemental Material - Walking a tightrope: Social support in early adulthood in resource-constrained South Africa Supplemental Material for Walking a tightrope: Social support in early adulthood in resource-constrained South Africa by Dorottya Hoór, Vuyiswa Nxumalo, Nuala McGrath, Janet Seeley, Maryam Shahmanesh and Guy Harling in Journal of Social and Personal Relationships.
