# Supplementary material for: Walking a tightrope: Social support in early adulthood in resource-constrained South Africa
Source: J Soc Pers Relat. 2025 May 22;42(9):2493–515. doi: 10.1177/02654075251337551 (PMC12316383; doi:10.1177/02654075251337551)
Supplement: Supplemental Material - Walking a tightrope: Social support in early adulthood in resource-constrained South Africa [file sj-pdf-1-spr-10.1177_02654075251337551.pdf]

*VIII. Supplementary materials*

**Supplementary Material 1**: Name generator questions in the Sixhumene baseline survey

Exchange based name generator questions

- Emotional support: “Who are the people with whom you could confide in or talk to about your life or your problems? For example, when you are feeling sad, anxious or upset”
- Informational support: “Who are the people who could give you information or advice in relation to an important life decision, such as health or employment problem, or moving to another place?”
- Financial support: “Who are the people you could talk to if you need help with money, for instance borrowing money or food, or getting jobs that pay?”
- Practical support: “Who are the people you could ask for help with doing things? This might include tasks inside or outside your home, or borrowing other small things?”
- Social companionship: “Who are the people you really enjoyed socialising with? This might include people who have visited you, or whom you visited, or with whom you went out somewhere.”

Name interpreter questions on support frequency

- Emotional support: “Over the past six months, how often have you received emotional support from this person, such as when you are feeling sad, anxious or upset?”

- Practical support: “Over the past six months, how often have you received help doing things from this person, such as tasks inside or outside your home or borrowing things?
- Informational support: “Over the past six months, how often have you received information or advice from this person, such as about a health or employment problem, or moving to another place?”
- Financial support: “Over the past six month, how often have you received help with money from this person, such as borrowing money or food, or getting a job that pays?”
- Social companionship: “Over the past six month, how often have you been socialising with this person, such as visiting them, or them visiting you, or going out somewhere with them?”
- Negative relation: “Over the past six month, how often has this person argued with you or criticised you in a humiliating way?”

**Supplementary Material 2:** Distribution of perceived support scores

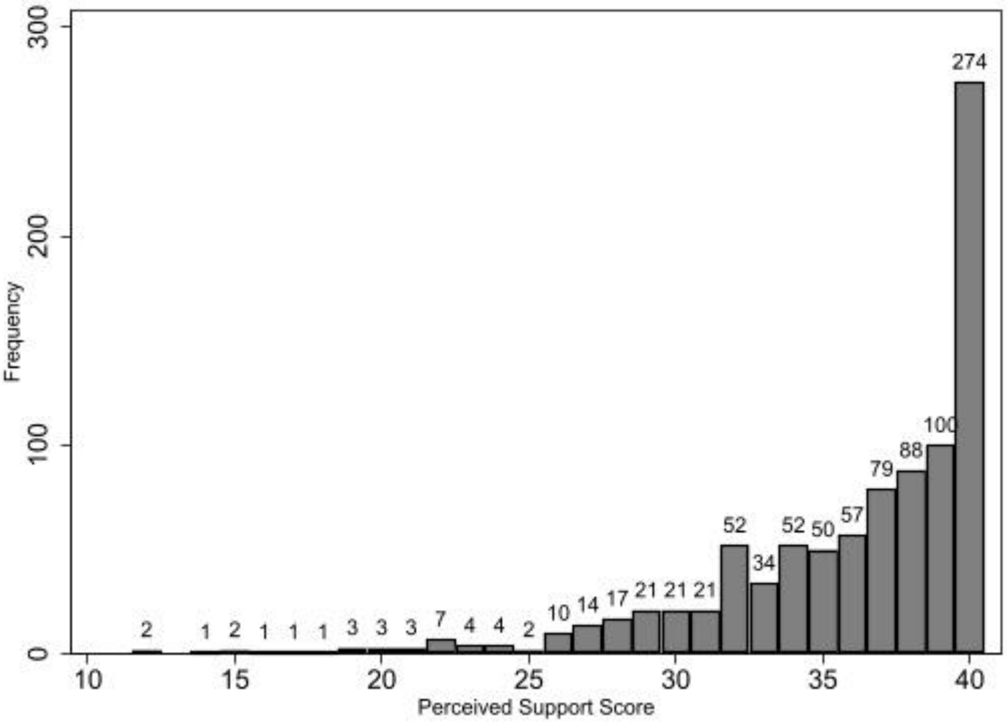

**Supplementary Material 3:** Demographic and network characteristics by age

| | Total<br>N=931 | 16-20<br>N=579 | 20-29<br>N=352 | p-value |
|---|---|---|---|---|
| Sex | | | | 0.003 |
| Male | 427 (40.0%) | 288 (49.7%) | 138 (39.7%) | |
| Female | 502 (54.0%) | 291 (50.3%) | 211 (60.3%) | |
| Age on interview date from verified DOB | 19 (17-22) | 17 (17-19) | 23 (22-24) | <0.001 |
| Presently lives with: Family | | | | 0.16 |
| No | 21 (2.3%) | 10 (1.7%) | 11 (3.1%) | |
| Yes | 910 (97.7%) | 569 (98.3%) | 340 (96.9%) | |
| Ever married | | | | 0.44 |
| No | 928 (99.9%) | 578 (99.8%) | 350 (100.0%) | |
| Yes | 1 (0.1%) | 1 (0.2%) | 0 (0.0%) | |
| Currently in school | | | | <0.001 |
| No | 453 (48.7%) | 138 (23.8%) | 315 (89.7%) | |
| Yes | 477 (51.3%) | 441 (76.2%) | 36 (10.3%) | |
| Highest completed level of education | | | | <0.001 |
| Primary school | 23 (2.5%) | 18 (3.1%) | 5 (1.4%) | |
| Secondary school | 571 (61.4%) | 452 (78.1%) | 119 (33.9%) | |
| Matriculation | 300 (32.3%) | 100 (17.3%) | 200 (57.0%) | |
| Certificate | 13 (1.4%) | 2 (0.3%) | 11 (3.1%) | |
| Diploma | 13 (1.4%) | 4 (0.7%) | 9 (2.6%) | |
| Bachelors degree | 9 (1.0%) | 3 (0.5%) | 6 (1.7%) | |
| Honors, Masters or higher | 1 (0.1%) | 0 (0.0%) | 1 (0.3%) | |
| Currently employed | | | | <0.001 |
| Full-time | 15 (1.6%) | 2 (0.3%) | 13 (3.7%) | |
| Part-time | 81 (8.7%) | 20 (3.5%) | 61 (17.4%) | |
| Not working | 834 (89.7%) | 557 (96.2%) | 276 (78.9%) | |
| **Network size** | | | | |
| Overall | 3.09 (1.37) | 3.19 (1.37) | 2.93 (1.35) | 0.004 |
| Emotional | 2.65 (1.56) | 2.75 (1.56) | 2.50 (1.56) | 0.019 |
| Practical | 1.86 (1.28) | 1.90 (1.30) | 1.80 (1.25) | 0.28 |
| Informational | 2.22 (1.65) | 2.23 (1.68) | 2.21 (1.60) | 0.87 |
| Financial | 1.82 (1.37) | 1.84 (1.33) | 1.79 (1.43) | 0.57 |
| Social companionship | 2.19 (1.58) | 2.27 (1.62) | 2.05 (1.52) | 0.044 |
| Negative | 0.57 (1.03) | 0.60 (1.06) | 0.51 (0.96) | 0.18 |
| Multiplexity | 3.29 (1.17) | 3.26 (1.12) | 3.33 (1.23) | 0.36 |
| Gender homophily (% of same-gender alters) | 68.4% | 69.6% | 66.4% | 0.11 |
| **Network composition** (mean proportion of networks) | | | | |
| Friend | 30.8% | 32.8% | 27.6% | 0.014 |
| Co-resident mother | 17.8% | 19.5% | 15.0% | 0.003 |
| Mother outside household | 3.8% | 4.5% | 2.6% | 0.009 |
| Co-resident father | 2.1% | 2.5% | 1.5% | 0.065 |
| Father outside household | 1.9% | 2.3% | 1.4% | 0.11 |
| Co-resident other family | 20.9% | 19.9% | 22.6% | 0.14 |
| Other family outside household | 11.8% | 11.1% | 12.9% | 0.21 |
| Romantic partner | 10.8% | 7.4% | 16.5% | <0.001 |

| | | | | |
|---|---|---|---|---|
| **Parental support** | | | | |
| Any parent (resident or not) | | | | <0.001 |
| none | 369 (39.6%) | 191 (33.0%) | 178 (50.6%) | |
| one parent | 465 (49.9%) | 310 (53.5%) | 155 (44.0%) | |
| both parents | 97 (10.4%) | 78 (13.5%) | 19 (5.4%) | |
| Co-resident parents | | | | <0.001 |
| none | 450 (48.3%) | 248 (42.8%) | 202 (57.4%) | |
| one parent | 447 (48.0%) | 305 (52.7%) | 142 (40.3%) | |
| both parents | 34 (3.7%) | 26 (4.5%) | 8 (2.3%) | |
| Co-resident mothers | 451 (48.4%) | 309 (53.4%) | 142 (40.3%) | <0.001 |
| Co-resident fathers | 64 (6.9%) | 48 (8.3%) | 16 (4.5%) | 0.029 |
| Mother outside household | 115 (12.4%) | 86 (14.9%) | 29 (8.2%) | 0.003 |
| Father outside household | 63 (6.8%) | 47 (8.1%) | 16 (4.5%) | 0.035 |
| Mother (co-resident and not) | 532 (57.1%) | 371 (64.1%) | 161 (45.7%) | <0.001 |
| Father (co-resident and not) | 127 (13.6%) | 95 (16.4%) | 32 (9.1%) | 0.002 |
| | | | | |
| **Monthly support frequency** | | | | |
| Emotional | 54.4 (49.4) | 57.6 (49.9) | 49.1 (48.2) | 0.010 |
| Practical | 33.3 (30.7) | 35.2 (31.9) | 30.0 (28.4) | 0.012 |
| Informational | 33.5 (38.4) | 34.8 (38.7) | 31.3 (37.8) | 0.18 |
| Financial | 20.5 (25.7) | 23.2 (27.1) | 16.0 (22.5) | <0.001 |
| Social companionship | 35.0 (38.3) | 38.0 (39.5) | 30.1 (35.6) | 0.002 |
| Conflict | 6.10 (16.12) | 6.85 (16.64) | 4.87 (15.18) | 0.069 |

Data are presented as mean (SD) or median (IQR) for continuous measures, and n (%) for categorical measures.

**Supplementary Material 4:** Tie level characteristics (monthly support and mean age difference)

| | **Total** | **Male** | **Female** | **p-value** |
|---|---|---|---|---|
| | N=3,021 | N=1,385 | N=1,636 | |
| Emotional support | 16.8 (13.6) | 15.5 (13.7) | 17.9 (13.5) | <0.001 |
| Physical support | 10.3 (13.2) | 9.3 (12.7) | 11.1 (13.6) | <0.001 |
| Informational support | 10.3 (12.4) | 9.1 (11.8) | 11.4 (12.8) | <0.001 |
| Financial support | 6.3 (10.8) | 5.9 (10.5) | 6.7 (11.0) | 0.036 |
| Social companionship | 10.8 (13.1) | 11.6 (13.3) | 10.1 (12.9) | 0.001 |
| Conflict | 1.9 (6.4) | 2.0 (6.5) | 1.8 (6.4) | 0.40 |
| Total support | 54.6 (41.9) | 51.4 (40.1) | 57.3 (43.3) | <0.001 |
| Ego-alter age difference | 9.5 (13.1) | 9.1 (13.4) | 9.9 (12.9) | 0.085 |

Data are presented as mean (SD).

**Supplementary Material 5**: *Multi-level ordinal logistic models of support frequencies by ego gender*

| | Emotional support | | | | | |
|---|---|---|---|---|---|---|
| | Male | | Female | | Chi2 | p-value |
| | OR | CI | OR | CI | | |
| Co-resident mother | 7.26 | [4.18,12.61] | 8.67 | [4.69,16.01] | 0.30 | 0.581 |
| Co-resident father | 2.56 | [1.01,6.48] | 5.38 | [1.68,17.27] | 1.13 | 0.288 |
| Mother outside household | 0.92 | [0.44,1.96] | 2.76 | [1.19,6.41] | 4.33 | 0.037 |
| Father outside household | 0.68 | [0.28,1.70] | 0.96 | [0.33,2.79] | 0.26 | 0.608 |
| Other co-resident family | 2.40 | [1.54,3.75] | 3.51 | [2.04,6.02] | 1.66 | 0.198 |
| Other family outside household | 0.77 | [0.48,1.23] | 1.56 | [0.87,2.80] | 4.45 | 0.035 |
| Friend | 1 | [1.00,1.00] | 2.25 | [1.36,3.73] | 9.98 | 0.002 |
| Romantic partner | 1.76 | [1.00,3.10] | 3.02 | [1.71,5.34] | 2.16 | 0.142 |
| Network size | 2.28 | [1.95,2.66] | | | | |
| Ego-alter age difference | 1.00 | [0.99,1.01] | | | | |
| Ego age | 0.96 | [0.90,1.03] | | | | |
| Egos | 927 | | | | | |
| Alters | 2986 | | | | | |

OR: Odds Ratios ; 95% confidence intervals in brackets
Number of egos <929 and number of alters<3023 due to some missing ego, alter and tie characteristics

| Practical Support | | | | | | |
|---|---|---|---|---|---|---|
| | Male | | Female | | Chi2 | p-value |
| | OR | CI | OR | CI | | |
| Co-resident mother | 30.2 | [18.88,48.38] | 45.3 | [28.37,72.28] | 3.15 | 0.076 |
| Co-resident father | 19.3 | [9.01,41.30] | 11.7 | [4.70,29.10] | 0.84 | 0.360 |
| Mother outside household | 0.61 | [0.30,1.24] | 0.71 | [0.36,1.39] | 0.12 | 0.732 |
| Father outside household | 0.42 | [0.17,1.06] | 0.47 | [0.17,1.27] | 0.02 | 0.876 |
| Other co-resident family | 19.0 | [12.98,27.96] | 29.6 | [19.89,44.09] | 4.49 | 0.034 |
| Other family outside household | 1.27 | [0.85,1.89] | 0.96 | [0.63,1.47] | 1.18 | 0.278 |
| Friend | 1 | [1.00,1.00] | 0.95 | [0.68,1.33] | 0.08 | 0.782 |
| Romantic partner | 0.61 | [0.37,1.01] | 0.25 | [0.16,0.42] | 7.25 | 0.007 |
| | | | | | | |
| Network size | 1.09 | [1.01,1.18] | | | | |
| Ego-alter age difference | 0.99 | [0.98,1.00] | | | | |
| Ego age | 1.03 | [0.99,1.07] | | | | |
| Egos | 924 | | | | | |
| Alters | 2974 | | | | | |

OR: Odds Ratios; 95% confidence intervals in brackets
Number of egos <929 and number of alters<3023 due to some missing ego, alter and tie characteristics

| Informational Support | | | | | | |
|---|---|---|---|---|---|---|
| | Male | | Female | | Chi2 | p-value |
| | OR | CI | OR | CI | | |
| Co-resident mother | 5.52 | [3.45,8.82] | 10.8 | [6.48,18.15] | 6.83 | 0.009 |
| Co-resident father | 1.89 | [0.82,4.36] | 2.48 | [0.89,6.94] | 0.19 | 0.659 |
| Mother outside household | 1.52 | [0.77,2.99] | 1.75 | [0.86,3.57] | 0.10 | 0.750 |
| Father outside household | 1.04 | [0.46,2.34] | 1.06 | [0.39,2.86] | 0.0 | 0.982 |
| Other co-resident family | 2.50 | [1.70,3.67] | 5.13 | [3.25,8.08] | 8.62 | 0.003 |
| Other family outside household | 1.22 | [0.80,1.86] | 1.77 | [1.08,2.92] | 1.64 | 0.201 |
| Friend | 1 | [1.00,1.00] | 2.10 | [1.38,3.21] | 11.84 | 0.001 |
| Romantic partner | 1.51 | [0.96,2.38] | 1.97 | [1.22,3.19] | 0.77 | 0.380 |
| | | | | | | |
| Network size | 1.77 | [1.57,2.01] | | | | |
| Ego-alter age difference | 1.01 | [1.00,1.02] | | | | |
| Ego age | 1.05 | [1.00,1.11] | | | | |
| Egos | 927 | | | | | |
| Alters | 2984 | | | | | |

OR: Odds Ratios; 95% confidence intervals in brackets
Number of egos <929 and number of alters<3023 due to some missing ego, alter and tie characteristics

| Financial Support | | | | | | |
|---|---|---|---|---|---|---|
| | Male | | Female | | Chi2 | p-value |
| | OR | CI | OR | CI | | |
| Co-resident mother | 7.65 | [4.84,12.10] | 6.37 | [4.10,9.90] | 0.66 | 0.417 |
| Co-resident father | 2.02 | [0.94,4.33] | 4.38 | [1.76,10.90] | 1.99 | 0.158 |
| Mother outside household | 2.60 | [1.39,4.87] | 2.91 | [1.54,5.53] | 0.08 | 0.776 |
| Father outside household | 2.32 | [1.11,4.85] | 2.97 | [1.23,7.17] | 0.21 | 0.646 |
| Other co-resident family | 1.83 | [1.26,2.65] | 1.94 | [1.32,2.84] | 0.07 | 0.792 |
| Other family outside household | 1.48 | [0.98,2.22] | 1.25 | [0.80,1.93] | 0.44 | 0.506 |
| Friend | 1 | [1.00,1.00] | 1.14 | [0.80,1.64] | 0.53 | 0.465 |
| Romantic partner | 0.64 | [0.38,1.08] | 4.08 | [2.71,6.12] | 37.88 | 0.000 |
| | | | | | | |
| Network size | 1.22 | [1.12,1.34] | | | | |
| Ego-alter age difference | 1.05 | [1.04,1.06] | | | | |
| Ego age | 1.01 | [0.97,1.05] | | | | |
| Egos | 923 | | | | | |
| Alters | 2976 | | | | | |

OR: Odds Ratios; 95% confidence intervals in brackets

Number of egos <929 and number of alters<3023 due to some missing ego, alter and tie characteristics

| Social Companionship | | | | | | |
|---|---|---|---|---|---|---|
| | Male | | Female | | Chi2 | p-value |
| | OR | CI | OR | CI | | |
| Co-resident mother | 0.98 | [0.61,1.59] | 1.25 | [0.76,2.05] | 0.88 | 0.348 |
| Co-resident father | 0.61 | [0.26,1.40] | 0.66 | [0.23,1.92] | 0.02 | 0.895 |
| Mother outside household | 0.038 | [0.02,0.08] | 0.091 | [0.04,0.18] | 3.43 | 0.064 |
| Father outside household | 0.039 | [0.02,0.09] | 0.029 | [0.01,0.08] | 0.21 | 0.645 |
| Other co-resident family | 0.69 | [0.47,1.02] | 1.06 | [0.69,1.62] | 3.08 | 0.079 |
| Other family outside household | 0.15 | [0.10,0.24] | 0.14 | [0.09,0.23] | 0.08 | 0.771 |
| Friend | 1 | [1.00,1.00] | 0.42 | [0.28,0.62] | 18.5 | 0.000 |
| Romantic partner | 0.25 | [0.16,0.40] | 0.19 | [0.12,0.29] | 1.11 | 0.292 |
| | | | | | | |
| Network size | 1.76 | [1.57,1.97] | | | | |
| Ego-alter age difference | 0.99 | [0.98,1.00] | | | | |
| Ego age | 0.99 | [0.95,1.04] | | | | |
| Egos | 926 | | | | | |
| Alters | 2985 | | | | | |

OR: Odds Ratios; 95% confidence intervals in brackets

Number of egos <929 and number of alters<3023 due to some missing ego, alter and tie characteristics

| | Conflict | | | | | |
|---|---|---|---|---|---|---|
| | Male | | Female | | Chi2 | p-value |
| | OR | CI | OR | CI | | |
| Co-resident mother | 5.68 | [2.60,12.41] | 4.36 | [1.90,10.01] | 0.48 | 0.487 |
| Co-resident father | 2.39 | [0.68,8.39] | 0.87 | [0.12,6.39] | 0.83 | 0.363 |
| Mother outside household | 0.43 | [0.13,1.43] | 0.079 | [0.02,0.33] | 3.82 | 0.051 |
| Father outside household | 0.074 | [0.01,0.42] | 0.21 | [0.03,1.57] | 0.68 | 0.409 |
| Other co-resident family | 0.92 | [0.47,1.81] | 0.85 | [0.39,1.88] | 0.03 | 0.855 |
| Other family outside household | 0.40 | [0.18,0.86] | 0.16 | [0.06,0.42] | 2.59 | 0.108 |
| Friend | 1 | [1.00,1.00] | 0.39 | [0.18,0.85] | 5.68 | 0.017 |
| Romantic partner | 2.56 | [1.06,6.19] | 0.46 | [0.19,1.08] | 9.56 | 0.002 |
| | | | | | | |
| Network size | 0.69 | [0.57,0.85] | | | | |
| Ego-alter age difference | 1.02 | [1.00,1.04] | | | | |
| Ego age | 0.92 | [0.84,1.01] | | | | |
| Egos | 927 | | | | | |
| Alters | 2983 | | | | | |

OR: Odds Ratios; 95% confidence intervals in brackets

Number of egos <929 and number of alters<3023 due to some missing ego, alter and tie characteristics
